# Supplementary material for: The crystal structure of a simian Foamy Virus receptor binding domain provides clues about entry into host cells
Source: Nat Commun. 2023 Mar 6;14:1262. doi: 10.1038/s41467-023-36923-0 (PMC9988990; doi:10.1038/s41467-023-36923-0)
Supplement: Supplementary file 1 — Supplementary information [file 41467_2023_36923_MOESM1_ESM.pdf]

Supplementary Tables and Figures

Table of Contents

Supplementary Tables and Figures **1**

Supplementary tables **2**

Table S1: X-ray crystallography data collection and refinement statistics. 2
Table S2: Secondary structure content in GII RBD 4
Table S3: Intramolecular interactions within GII RBD 5

Supplementary figures **7**

Figure S1: The fold of the FV RBD is maintained by hydrophobic and polar interactions 7
Figure S2: Mobile loops decorate the apex of the RBD 8
Figure S3: Comparison of glycosylated vs deglycosylated RBD structures 9
Figure S4: Comparison of the SFV RBD fold with that of the RBD of Orthoretroviruses 11
Figure S5: Intramolecular contacts between N8 sugar and RBD 12
Figure S6: Sequence conservation of FV Env 13
Figure S7: Functional features of FV EBD mapped onto the structure 17
Figure S8: AlphaFold 2 models of FV RBDs 18
Figure S9: FV RBD common core excludes a large portion of the upper subdomain 20
Figure S10: The upper domain loops show poor sequence conservation 21
Figure S11: RBD loops are functionally important for viral particles production, cell binding and infectivity 22
Figure S12: Recombinant RBD variants remain monomeric in solution 24
Figure S13: Flow cytometry gating strategy 25
Figure S14: Effect of mutations on FVV release and infectious titer 27
Figure S15: Structural basis for RBDjoin region being dispensable for binding to cells 28

References **29**

Supplementary tables

Table S1: X-ray crystallography data collection and refinement statistics.

	SFV GII RBD^D (native) data (PBD 8AEZ)	RBD^D (derivative) data	SFV GII RBD^G (native) data (PBD 8AIC)
**Data collection**			
Wavelength	0.9786	1.907	0.9786
Space group	$P3_221$	$P3_121$	$P6_1$
Cell dimensions			
a, b, c (Å)	99.5, 99.5, 120.6	99.6, 99.6, 120.9	123.6, 123.6, 191.6
a, b, g (°)	90, 90, 120	90, 90, 120	90, 90, 120
Resolution range (Å)	49.73 - 2.574 (2.666 - 2.574) [a]	46.06 - 3.171 (3.284 - 3.171)	46.73 - 2.8 (2.9 - 2.8)
Total reflections	453419 (44048)	238580 (24149)	875971 (87278)
Unique reflections	22363 (2191)	12192 (1165)	40619 (4041)
Completeness (%)	99.91 (99.50)	99.60 (96.75)	99.58 (99.14)
Redundancy	20.3 (20.1)	19.6 (20.1)	21.6 (21.6)
R_{merge}	0.2051 (0.9362)	0.1776 (0.9898)	0.199 (1.705)
R_{pim}	0.04701 (0.2117)	0.04098 (0.2256)	0.04364 (0.3719)
I/s (*I*)	10.10 (1.85)	20.30 (5.19)	13.35 (1.45)
$CC_{1/2}$	0.986 (0.846)	0.998 (0.917)	0.997 (0.689)
**Refinement**			
No. reflections	22355 (2190)	/	40615 (4040)
No. of reflections for R_{free}[b]	1052 (110)	/	2111 (208)
R_{work}/R_{free}	0.213/0.253	/	0.194/0.229
No. non-hydrogen atoms	2945	/	6086
Macromolecules	2711	/	5448
Ligands	203	/	531
Water	93	/	321
**Mean B value (Å^2)**			
Protein and sugar	73.88	/	65.44
Ligand/Ion	94.16	/	114.49
Water	60.68	/	55.18
**R.m.s. deviations**			
Bond lengths (Å)	0.012	/	0.004
Bond angles (°)	1.62	/	0.77
Ramachandran favored/outliers (%)	95.99/0.31	/	95.69/0.00

[a] Values in parentheses are for highest-resolution shell.
[b] The free set represents a random 5% of reflections not included in refinement

Table S2: Secondary structure content in GII RBD

Domain	Helical† (%)	β-strands (%)	Other‡ (%)
RBD	30	14	56
Lower	45	17	38
Upper	20	12	68

† - α- and 3_{10} helices
‡ - B, S, T, X

The secondary structure content was calculated using 2StrucCompare webserver [1] at https://2struccompare.cryst.bbk.ac.uk/index.php.

Table S3: Intramolecular interactions within GII RBD

The intramolecular interactions were analyzed by ProteinTools program https://proteintools.uni-bayreuth.de [2].

Van der Waals contacts in the SFV RBD			
Cluster #	Area (Å^2)	# of residues	Location (domain)
1	153	4	Lower
2	1002	22	
3	2543	51	Lower + upper
4	82	2	Upper
5	77	2	
6	86	2	

Polar contacts in the SFV RBD

Lower subdomain

Cluster	Donor - Acceptor	Distance (Å)
1	HIS225-ND1 -- GLN222-OE1	3.3
2	**ARG226-NH1 -- GLU337-OE1**	3.1
	**ARG226-NH2 -- GLU337-OE2**	2.4
3	HIS234-ND1 -- TYR323-OH	3.1
4	THR242-OG1 -- GLN492-OE1	3.4
5	HIS314-ND1 -- THR313-OG1	2.9
6	TYR327-OH -- ASP320-OD2	2.5
7	ASN331-ND2 -- ASN336-OD1	3.4
8	**LYS342-NZ -- GLU339-OE1**	3.3
	**ARG343-NE -- GLU339-OE2**	2.8
	**ARG343-NH2 -- GLU339-OE2**	3.2
9	ASN351-ND2 -- GLU502-OE1	2.5
	TYR551-OH -- GLU502-OE2	2.5
10	**LYS352-NZ -- GLU495-OE2**	3.2
	TYR497-OH -- GLU495-OE1	3.1
11	ASN368-ND2 -- ASN373-OD1	3.0

Upper subdomain

Cluster	Donor - Acceptor	Distance (Å)
12	GLN244-NE2 -- GLN491-OE1	2.6
	GLN491-NE2 -- SER488-OG	3.0
13	TYR267-OH -- ASP468-OD2	2.9
14	THR288-OG1 -- GLU442-OE1	2.9
	THR288-OG1 -- GLU442-OE2	3.4

	TYR456-OH -- GLU442-OE2	3.2
15	**ARG297-NH1 -- ASP402-OD2**	2.9
	**ARG297-NH2 -- ASP402-OD1**	2.6
	**ARG297-NH2 -- ASP402-OD2**	2.9
	SER397-OG -- ASP402-OD2	2.8
16	**ARG372-NH1 -- ASP378-OD2**	2.8
	**ARG372-NH2 -- ASP378-OD1**	2.9
	**ARG382-NH1 -- ASP378-OD1**	2.8
	SER375-OG -- ASP378-OD2	2.9
17	TRP399-NE1 -- ASP254-OD1	2.7
18	THR406-OG1 -- ASN409-OD1	3.4
19	**ARG407-NE -- GLU400-OE2**	3.1
20	**ARG433-NH2 – GLU464-OE2**	2.7
21	TRP435-NE1 -- ASN462-OD1	3.0
22	**ARG436-NH2 – ASP254-OD1**	2.6
23	THR452-OG1 -- ASP450-OD1	2.9
24	SER461-OG -- GLU439-OE2	2.5
25	GLN482-NE2 -- SER473-OG	3.1
26	**ARG537-NE -- GLU384-OE1**	3.0
	ARG537-NH1 -- TYR269-OH	3.4
	**ARG537-NH2 -- GLU384-OE2**	2.8
	**ARG537-NH2 -- ASP274-OD2**	2.8
	TYR269-OH -- ASP274-OD1	2.6
	TYR275-OH -- GLU384-OE2	2.6

Supplementary figures

Figure S1: The fold of the FV RBD is maintained by hydrophobic and polar interactions

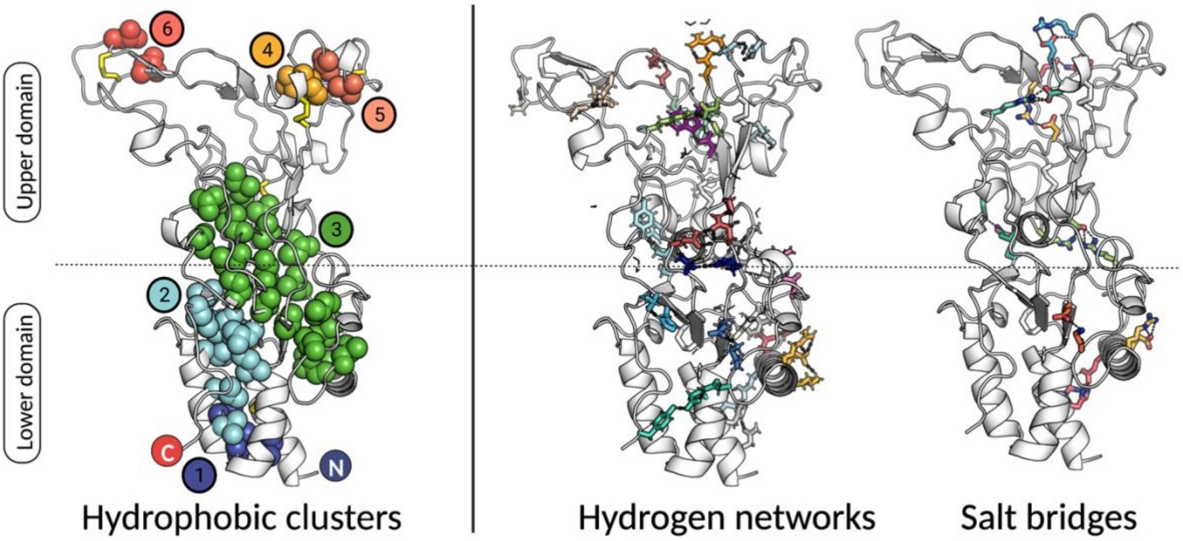

Figure S1 legend: The FV RBD core is formed by the hydrophobic residues grouped in 6 clusters - 2 in the lower subdomain (clusters #1 and #2), 3 in the upper subdomain (clusters #4, #5, #6), and the largest hydrophobic cluster (BSA=2451 Å^2 with 51 participating residues; cluster #3 shown in green) running in the direction of the longer axis of the RBD and containing residues from both domains. There are 24 networks of residues whose side chains contribute to 43 hydrogen bonds, with 21 charged residues forming 9 salt bridges. The area of the hydrophobic interfaces in the lower subdomain is about 6 times larger than in the upper subdomain, while the hydrogen bonds and salt bridges are more prevalent in the upper subdomain. The full list of intramolecular interactions and relevant details are given in Table S3. The intramolecular interactions were analyzed by ProteinTools program https://proteintools.uni-bayreuth.de [2].

Figure S2: Mobile loops decorate the apex of the RBD

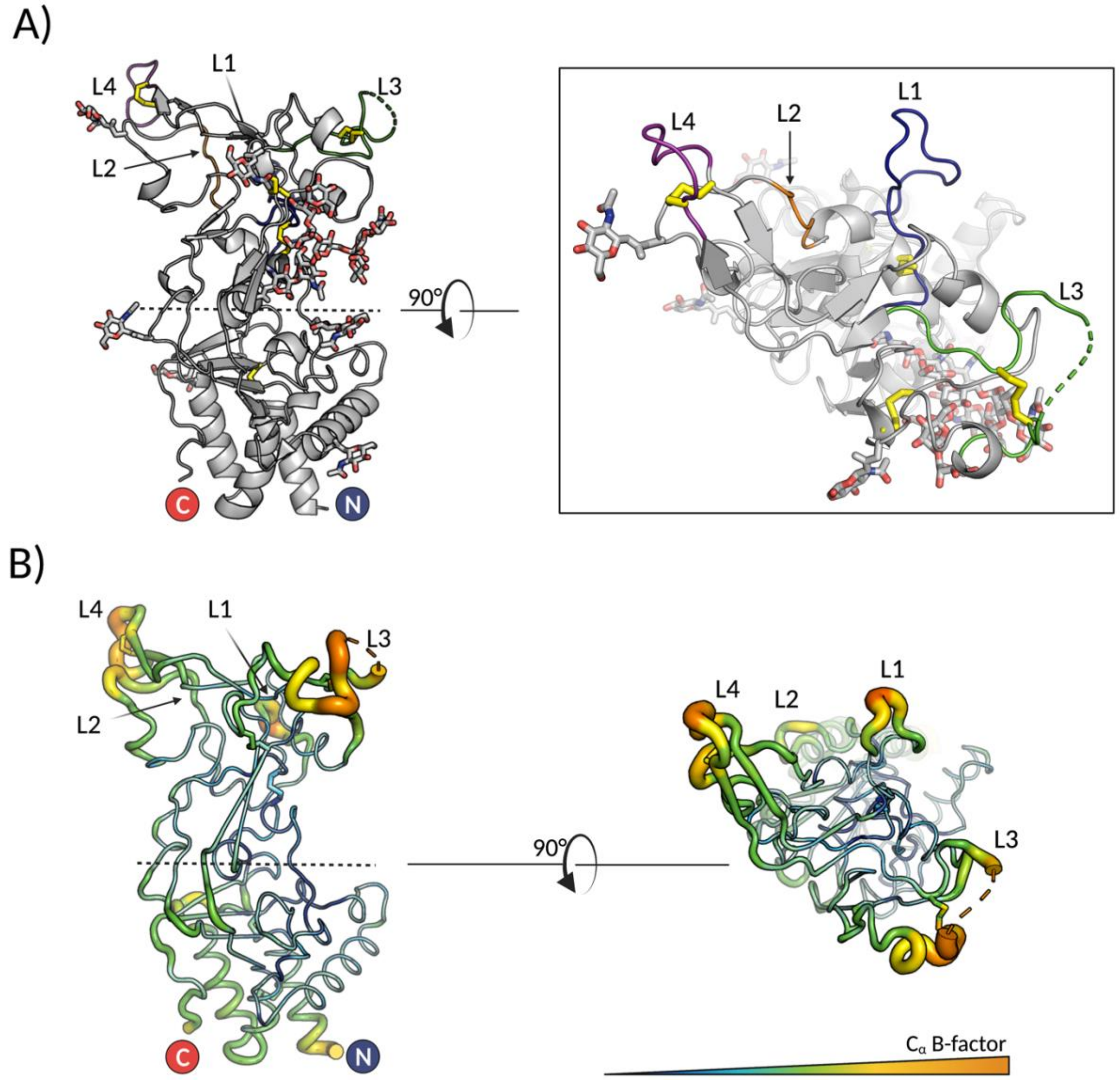

Figure S2 legend: A) The upper subdomains loops are designated as follows: loop 1 (L1, residues 253-270, connecting β2 and η1) in blue, loop 2 (L2, residues 276-281, connecting η1 and β3) in orange, loop 3 (L3, residues 414-436, connecting α5 and β9) in green, and loop 4 (L4, residues 446-453, connecting β10 and β11) in dark purple. **B)** The RBD structure is shown in 'tube' presentation to illustrate the mobility, with the more flexible regions shown as thicker tubes. Coloring scheme corresponds to the C_α atomic B-factors (low to high B factors shown in blue to orange spectrum). The images were generated in Pymol [3].

Figure S3: Comparison of glycosylated vs deglycosylated RBD structures

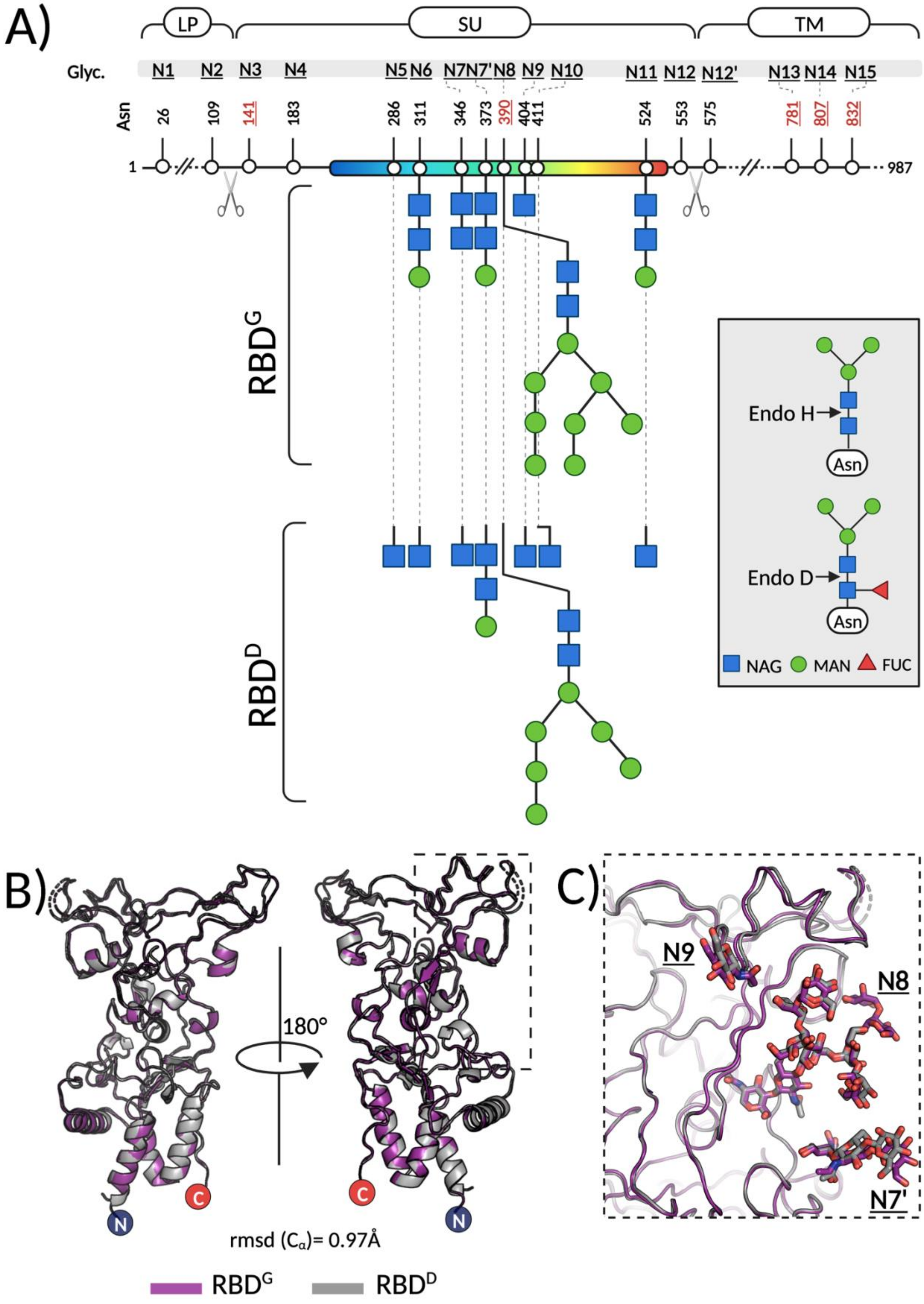

Figure S3 legend: A) Schematic representation of SFV Env and the 17 predicted N-glycosylation sites, labeled as N1 to N15 [4]. The sites that are 100% conserved in all FV Envs (Asn^{141} (N3), Asn^{390} (N8), Asn^{781} (N13), Asn^{807} (N14), Asn^{832} (N15)) are indicated with red underscored numbers. The two furin sites are represented by scissors. LP, SU and TM are the abbreviations for the leader peptide, surface subunit and transmembrane subunit, respectively. The sugar residues, N-acetyl glucosamine (NAG) and mannose (MAN) that could be resolved in RBD^D or RBD^G are shown.

The N-linked oligosaccharide core is shown in the grey inlet, with the cleavage sites indicated for the EndoD and EndoH glycosidases. A fraction of proteins expressed in insect cells contains an α1-6 fucose bound to the first NAG, rendering the sugar sensitive to cleavage by EndoD, but resistant to EndoH. Thus, both EndoD and EndoH were used for deglycosylation of the recombinant RBD. The figure was created in Biorender.com.

B) Superposition of the RBD^G (purple) and RBD^D (grey) structures done in Pymol [3]. For clarity reasons, only polypeptide chains are displayed. The boxed region on the right structure is magnified in panel C)

C) The polypeptide chain (in wire model) and sugars N7', N8 and N9 (shown as sticks) are displayed to illustrate absence of local changes induced by the sugars in RBD^G. Only molecule A is shown.

Figure S4: Comparison of the SFV RBD fold with that of the RBD of Orthoretroviruses

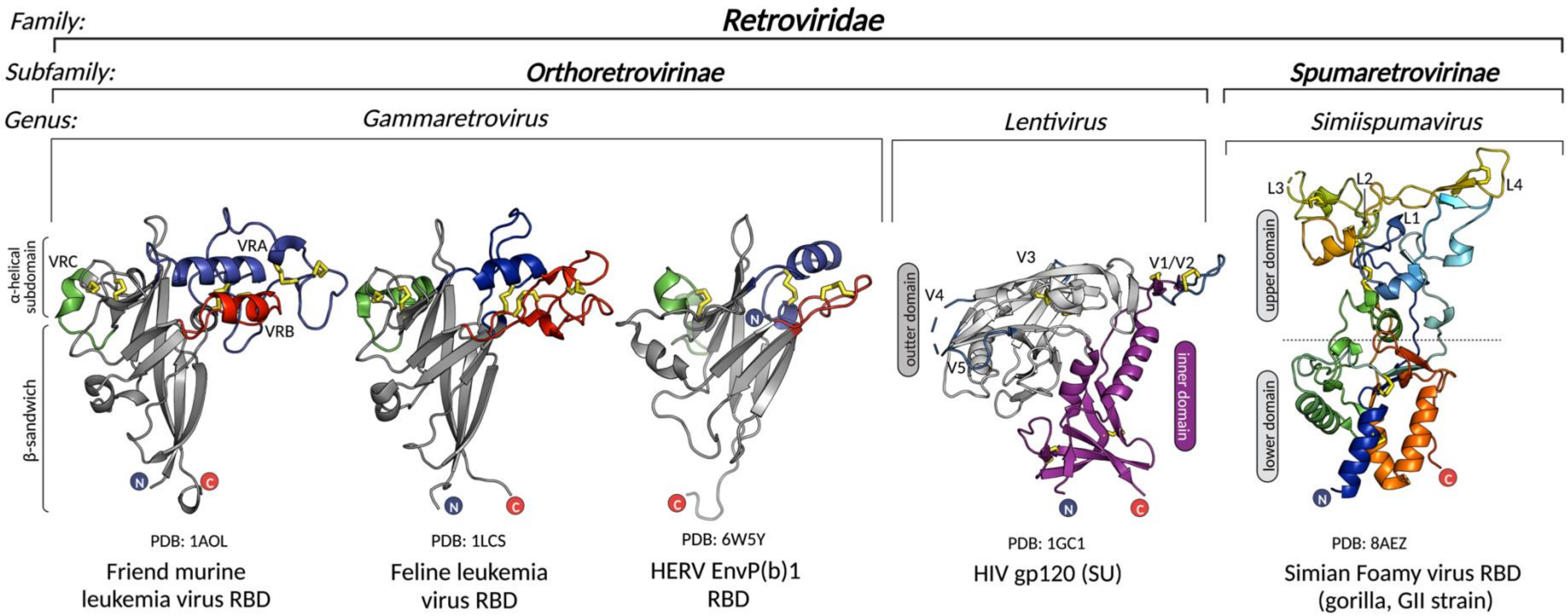

Figure S4 legend: Structures of RBDs from gammaretroviruses and of SU from HIV (lentivirus) are shown to illustrate a lack of structural homology between the RBDs from 3 different genera of retroviruses.

Figure S5: Intramolecular contacts between N8 sugar and RBD

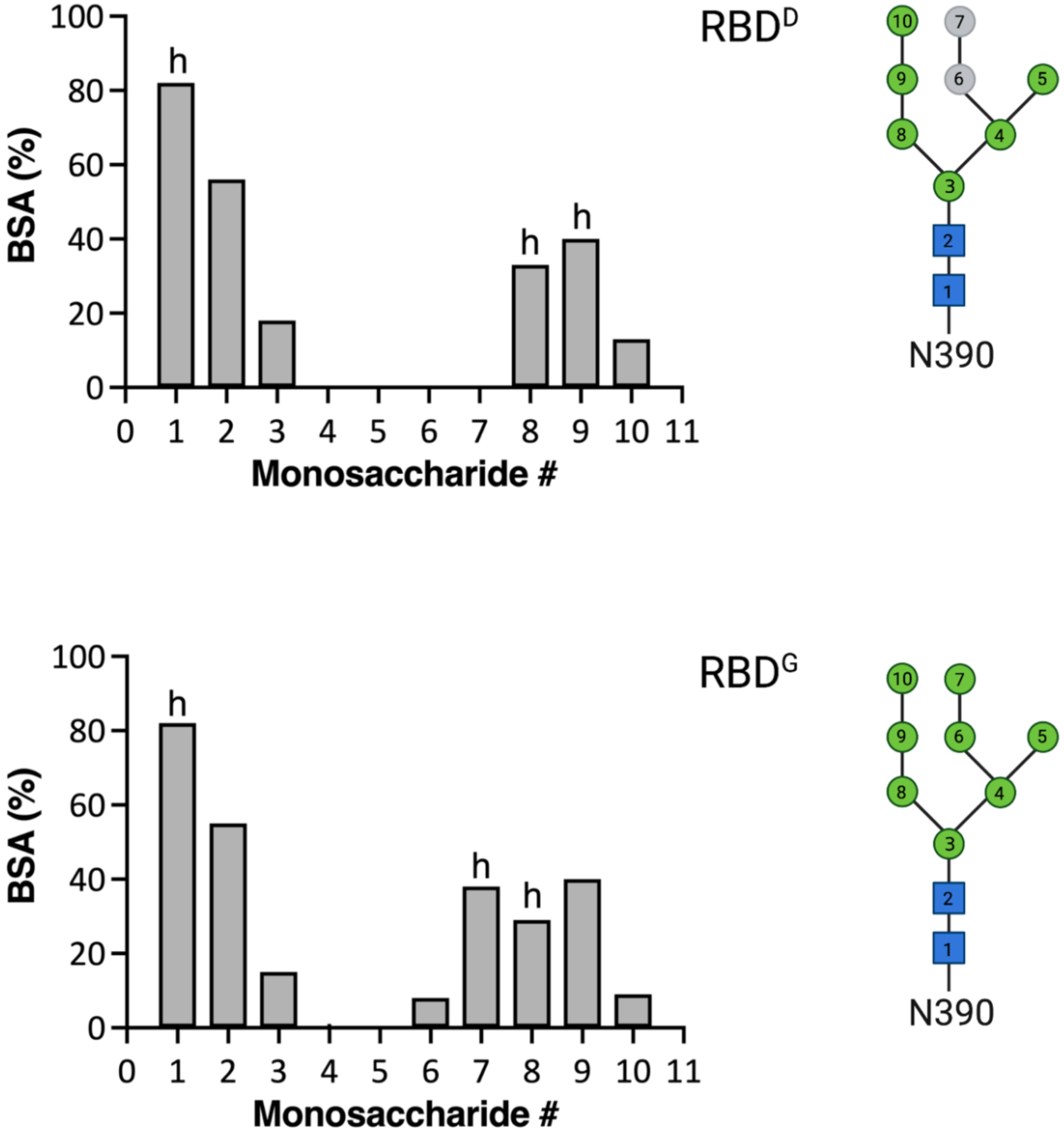

Figure S5: The buried surface area (BSA) for each sugar residue was calculated as a percent of the total surface area (Å^2) in ePISA [10] and plotted. Sugars that establish hydrogen bonds with the amino acids are indicated with letter ‘h’. Sugars 6 and 7 are colored in grey for RBD^D because they were not resolved in the structure.

Figure S6: Sequence conservation of FV Env

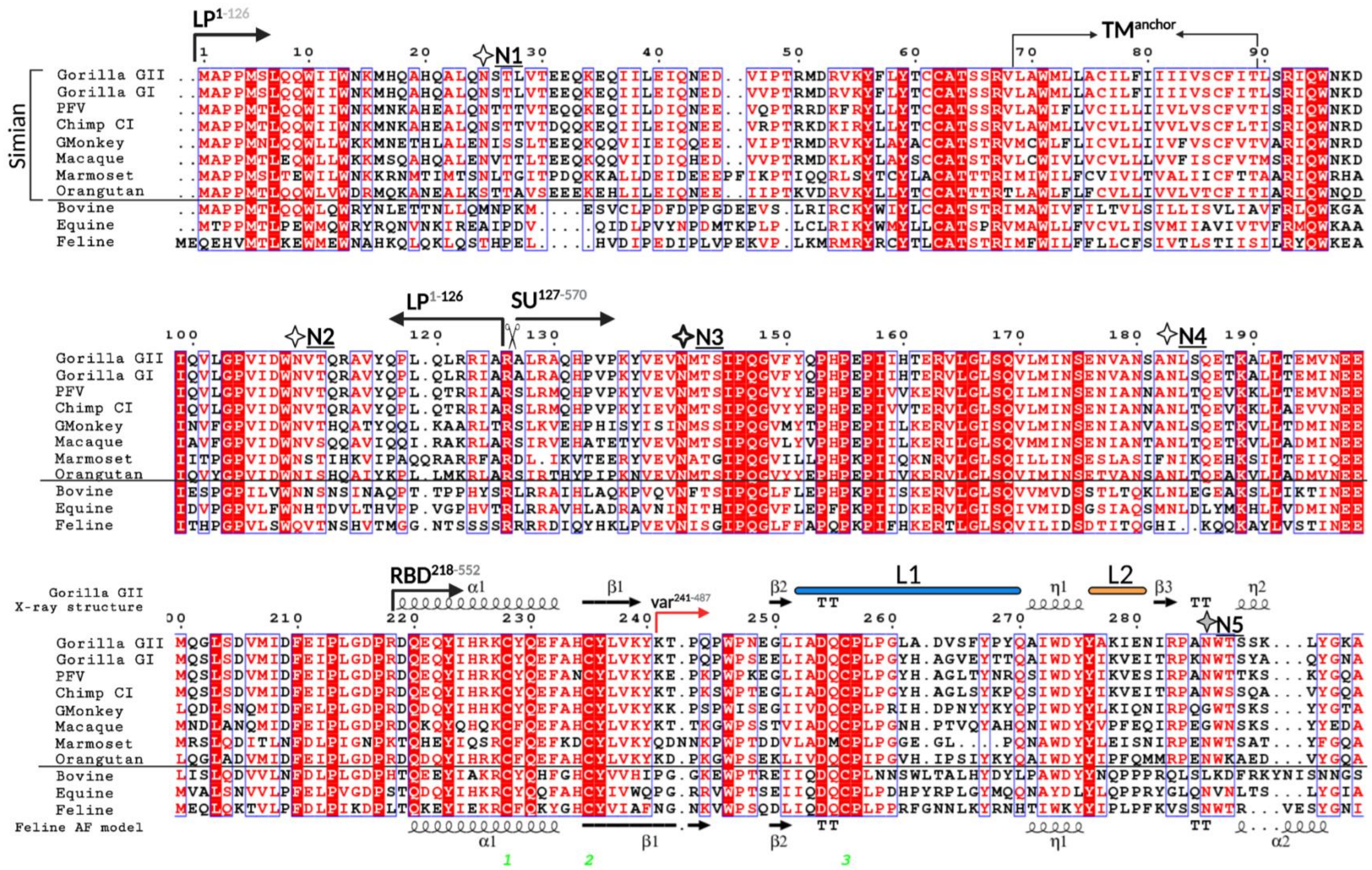

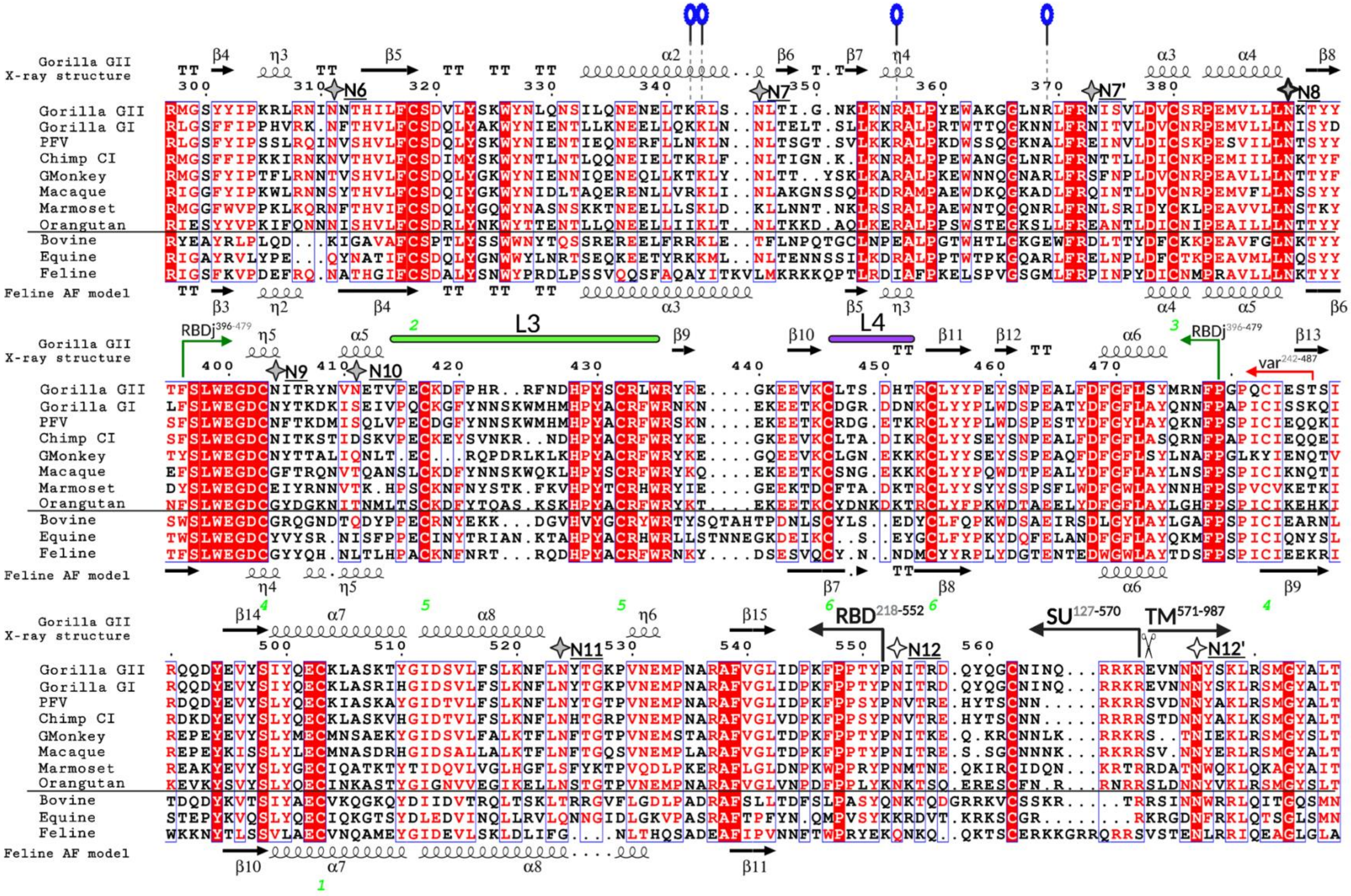
Gorilla GII
X-ray structure
Gorilla GII
Gorilla GI
PFV
Chimp CI
GMonkey
Macaque
Marmoset
Orangutan
Bovine
Equine
Feline
Feline AF model
N6
N7
N7'
N8
N9
N10
N11
N12
N12'
L3
L4
RBDj396-479
var242-487
RBD218-552
SU127-570
TM571-987

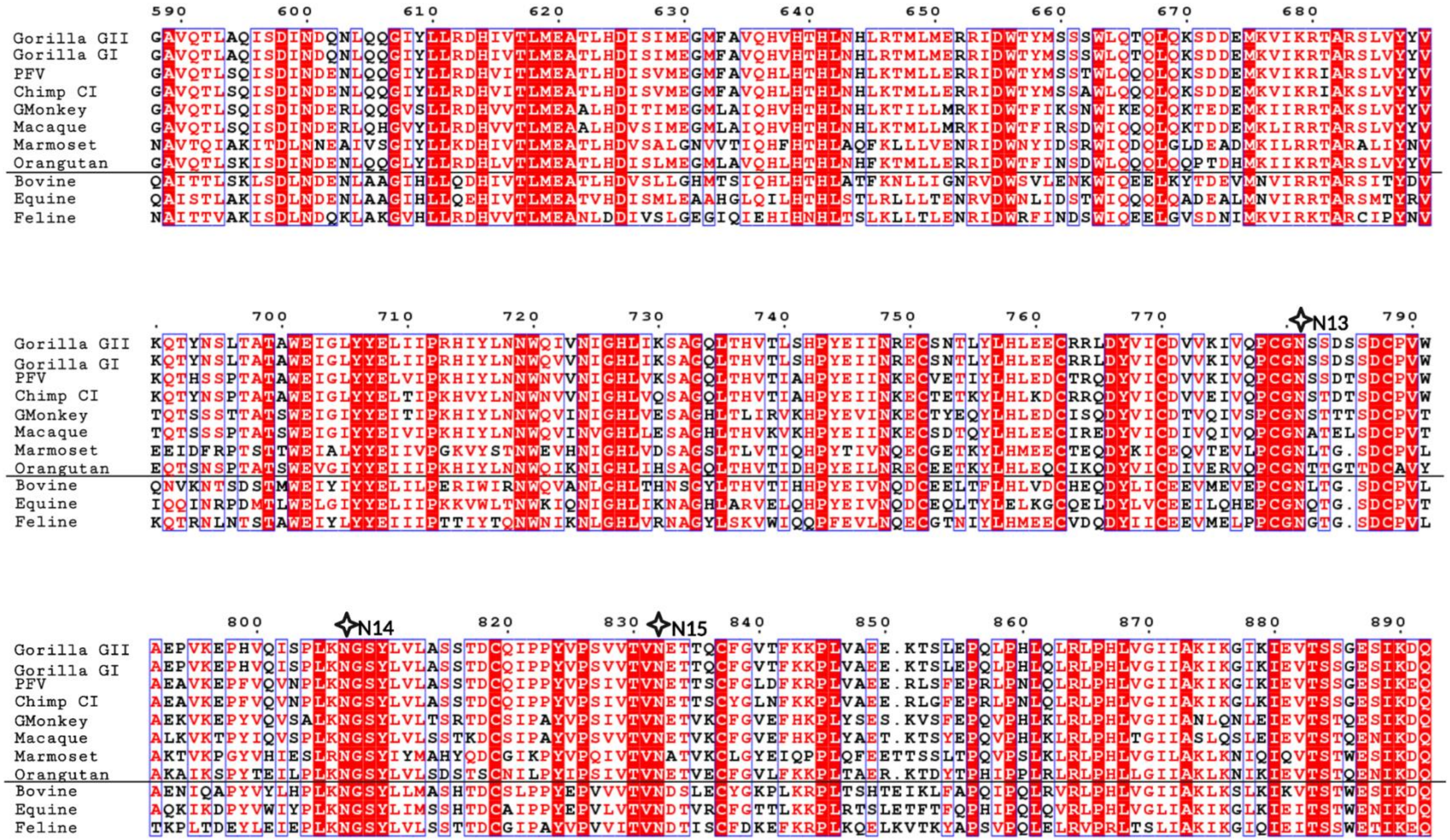
590
600
610
620
630
640
650
660
670
680
700
710
720
730
740
750
760
770
N13
790
800
N14
820
830
N15
840
850
860
870
880
890
Gorilla GII
Gorilla GI
PFV
Chimp CI
GMonkey
Macaque
Marmoset
Orangutan
Bovine
Equine
Feline

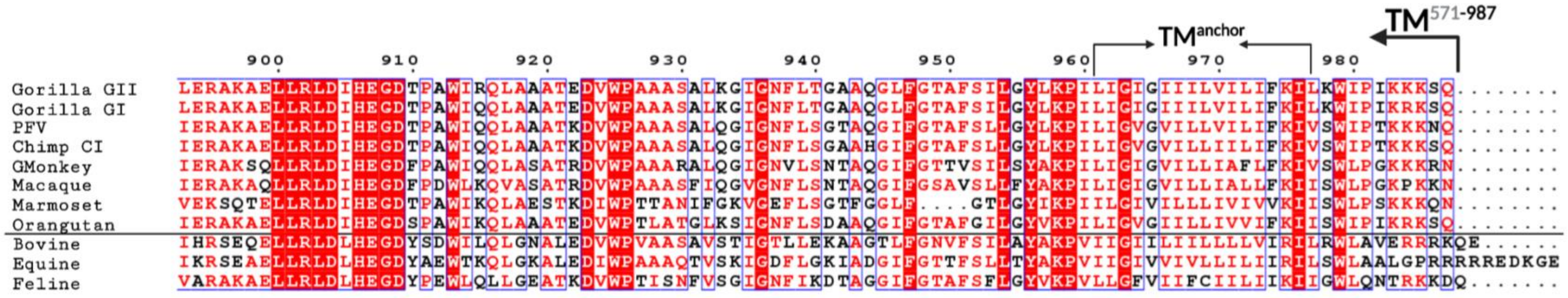

Figure S6 legend: Sequences corresponding to 11 FV Env were aligned in Clustal Omega [5] and the alignment was plotted with ESPript https://espript.ibcp.fr [6], with colors that indicate % identity (white letter, red background 100% identical; red letters, white background >70% identity; black letters, white background, <70% identity). The black, horizontal line separates simian from other FVs.

The secondary structure elements corresponding to the SFV RBD X-ray structure and the AF2 model for the feline FV RBD are plotted above and below the alignment, respectively. The N-linked glycosylation sites are indicated with stars. The already established nomenclature for the N-glycosylation sites (N1 to N15) is applied. The strictly conserved N-glycosylation sites are outlined with a thicker border, and the sites that carry sugars, which could be resolved in our structure, have grey filling. The residues interacting with heparan-sulfate are marked with blue ovals above the alignment. Loops 1-4 are indicated with bars above the alignment and labeled as L1-L4, using the same color code as in Fig. S2. The boundaries for the LP, SU, RBD, TM subunit are shown, as well as for the RBD variable and RBDjoin regions (the numbering corresponds to that of gorilla SFV Env, GII-K74 genotype). To distinguish the TM subunit from the TM domain, which is the region spanning the membrane, the latter is referred to as the TM^{anchor}. The two furin sites are indicated with the scissors icon.

The Env sequences used in the alignment were obtained from public databases and with following accession numbers: SFVggo_huBAK74 (GII-K74, genotype II gorilla SFV, GenBank: AFX98090.1), SFVggo_huBAD468 (GI-D468, genotype I gorilla SFV; GenBank: AFX98095.1), SFVpsc_huHSRV13 (CI-PFV, known as Prototype Foamy Virus genotype I Eastern chimpanzee SFV; GenBank: AQM52259.1), SFVcpz (genotype I Western chimpanzee; UniProtKB/Swiss-Prot: Q87041.1), SFVcae_LK3 (Genotype II African green monkey SFV; NCBI Reference: YP_001956723.2), SFVmcy_FV21 (genotype I macaque SFV; UniProtKB/Swiss-Prot: P23073.3), SFVcja_FXV (Marmoset FV; GenBank: GU356395.1), SFVppy_bella (Orangutan SFV; GenBank: CAD67563.1), BFVbta_BSV11 (Bovine FV; NCBI Reference: NP_044930.1), EFVeca_1 (Equine FV; GenBank: AAF64415.1), and FFVfca_FUV7 (Feline FV; UniProtKB/Swiss-Prot: O56861.1). Genotypes I and II have been defined for gorilla, chimpanzee, green monkey and macaque FVs [7,8,9].

Figure S7: Functional features of FV EBD mapped onto the structure

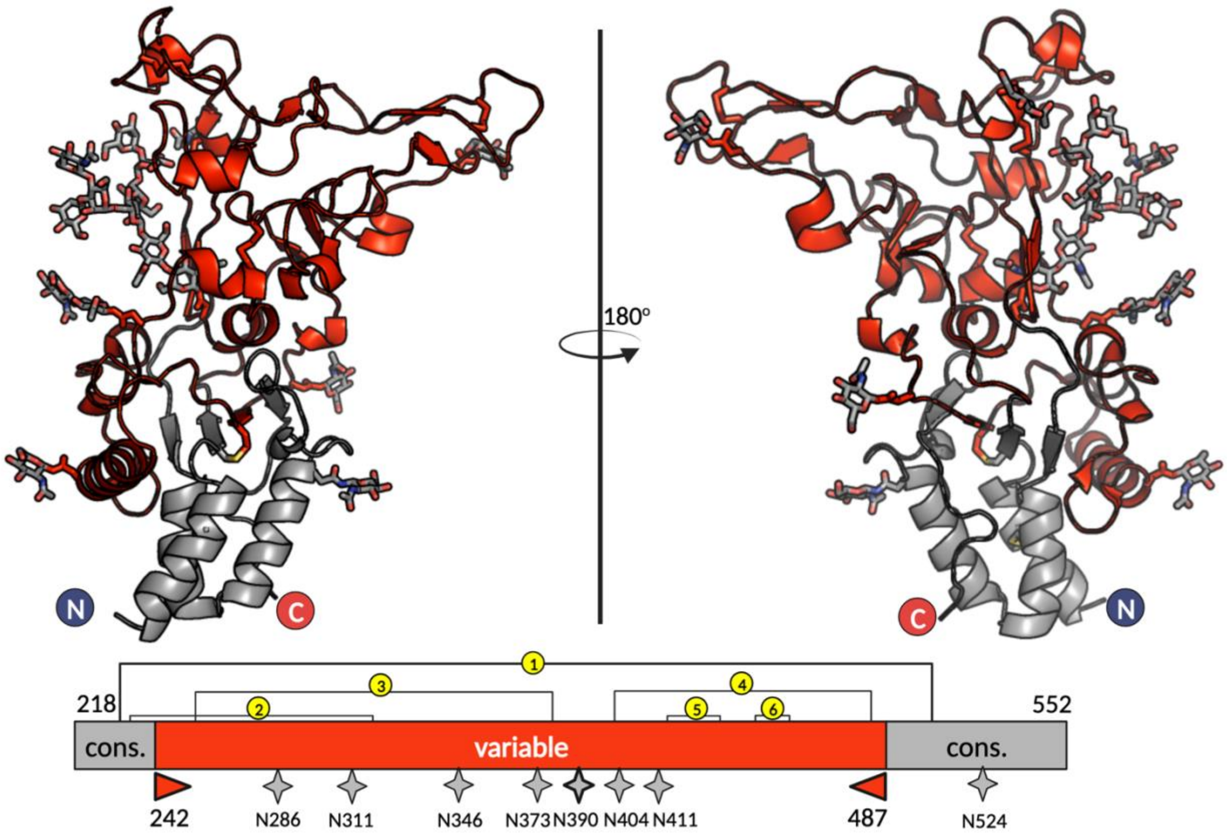

Figure S7: Functional features plotted on the RBD structure. The conserved 'RBD^{cons}' (residues 218-241 and 488-552) and variable regions 'RBD^{var}' (residues 242-487) are plotted on the X-ray structure of gorilla SFV RBD and colored in light grey and red, respectively. The glycosylation sites are indicated with the stars on the bottom.

Figure S8: AlphaFold2 models of FV RBDs

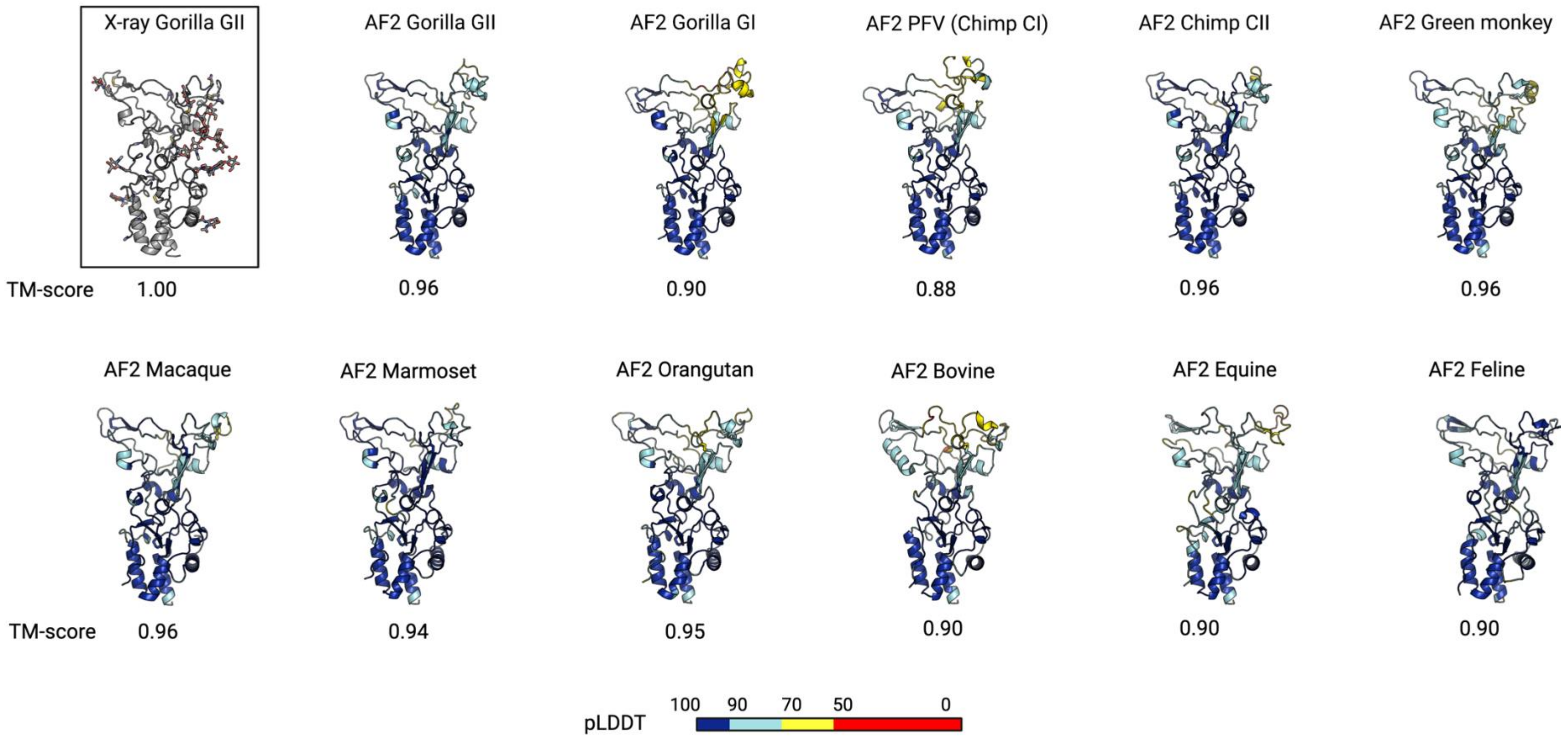

Figure S8 legend: AF2 prediction program [11] was used for *ab initio* modeling of the FV RBDs. The structures are colored according to the per-residue confidence metric called ‘predicted local distance difference test’ (pLDDT). The pLDDT can have a value between 0 and 100, with the higher model confidence corresponding to the higher pLDDT number. pLDDT > 90 (rendered in blue on the panels) is the high accuracy cut-off, above which the backbone and rotamers are predicted with high confidence; values between 70 and 90 (cyan) correspond to the regions where the backbone conformation is correct; values between 50 and 70 (yellow) have low confidence and are not reliably predicted, and regions with pLDDT below 50 (red) should not be interpreted.

Structural superpositions of all AF2 models against each other were carried out using mTM-align server for multiple structural alignments12,13 available at https://yanglab.nankai.edu.cn/mTM-align/. Below each model is a ‘template modelling score’ (TM-score), which is a length-independent scoring function reflecting the similarity of two structures14. The TM-scores can take values between 0 and 1, with the higher TM-score indicating higher structural similarity. The indicated TM-scores correspond to the pairwise superimposition of each AF2 model onto the X-ray Gorilla GII RBD structure. The accession codes for the AF2 models, which have been deposited in the Model Archive database, are provided in the ‘Data Availability’ section.

Figure S9: FV RBD common core excludes a large portion of the upper subdomain

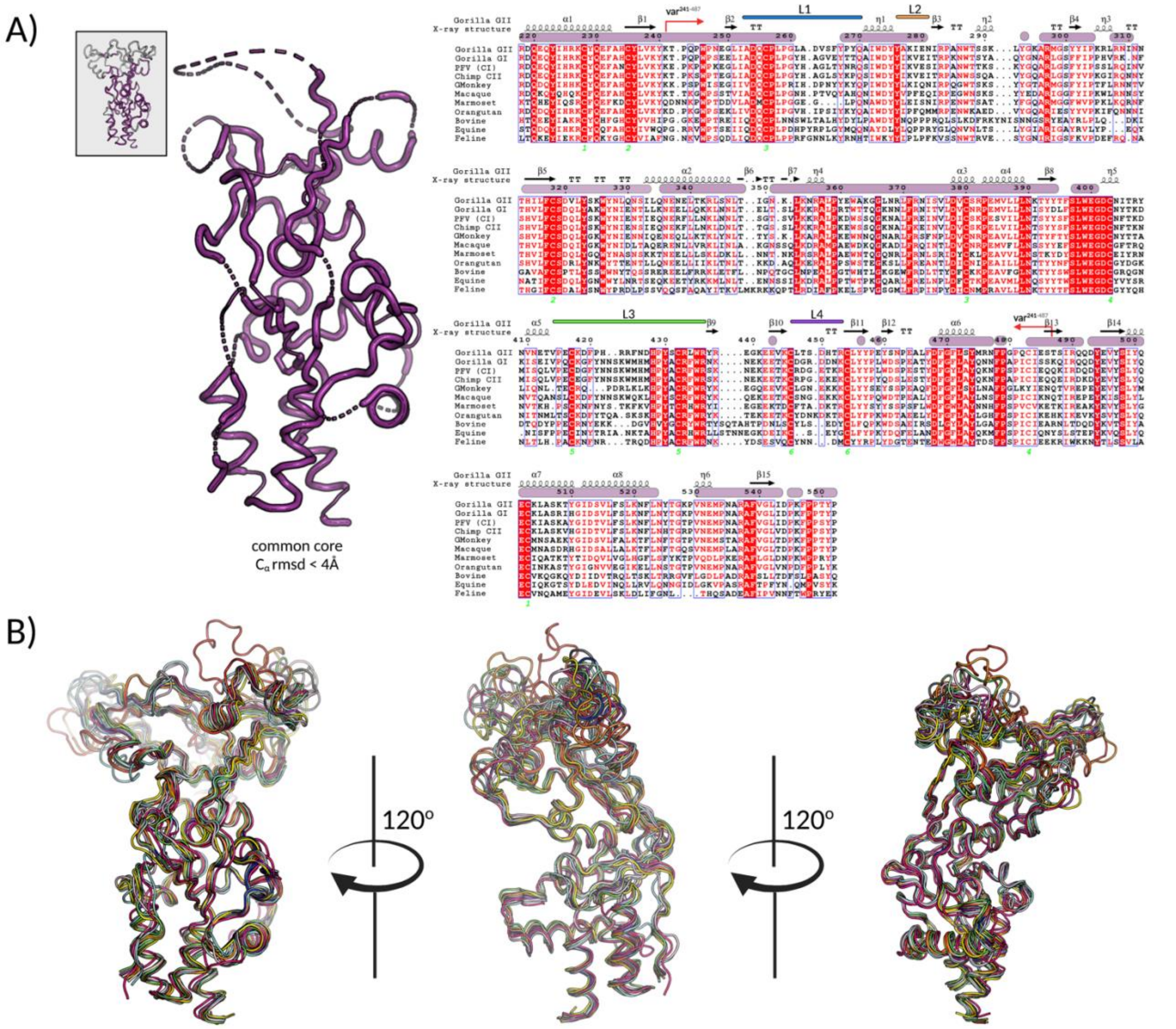

Figure S9 legend: **A)** Superposition of the RBD experimental structure and 11 AF2 models (Fig. S8) yielded a 'common core', model that includes the residues with Cα rmsd < 4Å for all pairwise superpositions. The regions containing residues with Cα rmsd > 4Å are indicated with dashed lines. The common core regions are highlighted with purple bars on top of the sequence alignment, which is colored using the same scheme as in Fig. S6. The small inlet in the upper left corner represents the entire RBD as a reference for comparison, with the common core colored in purple, and the remaining residues in grey. The structural and sequence alignments were carried out as described in Figs. S8 and S6, respectively.

B) Each RBD model is shown as a separate structure to illustrate contrast between the structurally conserved, common core residues, and the upper subdomains, whose loops are predicted to adopt different conformations.

Figure S10: The upper domain loops show poor sequence conservation

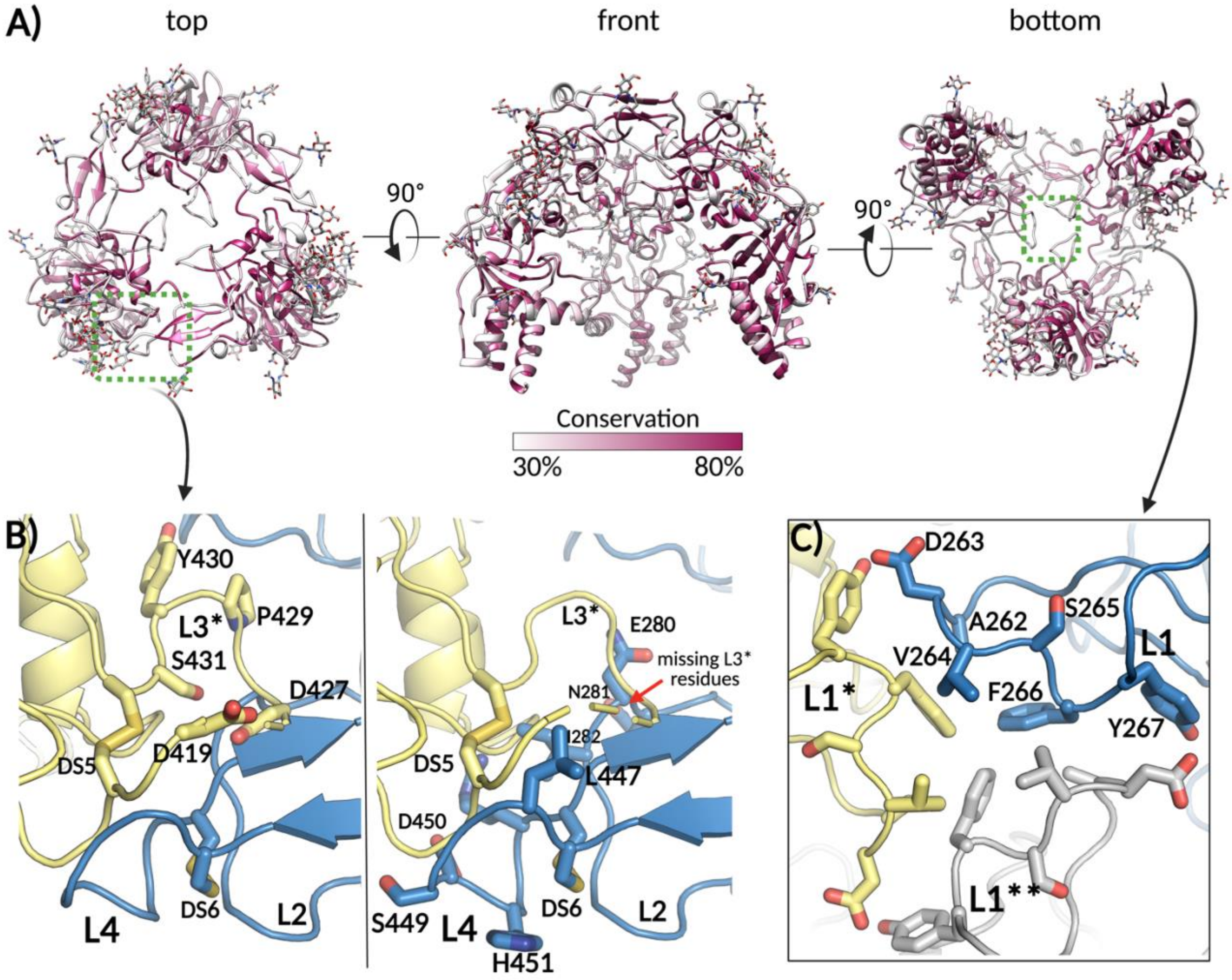

Figure S10 legend: A) Three SFV RBD protomers fitted in the density maps obtained for PFV Env [15], as shown in Fig. 4 are rendered by residue conservation. The % identity was calculated in Chimera [16] according to the sequence alignment shown in Fig. S6, and residues were colored with the white to maroon gradient as indicated with the color key. The residues showing less than 30% and more than 90% sequence identity are colored in solid white and maroon, respectively.

B) The region outlined with a green dashed-line rectangle in panel A (top view) is magnified, with the two protomers colored in yellow and blue, for clarity. Loops 4 and 2, and loop 3 of the neighboring protomer (L4, L2 and L3*, respectively) are indicated, along with the disulfide bonds DS5 and DS6, clamping the ends of the loops. On the left panels, residues from L3*, potentially forming contacts with L4 and L2 are displayed with their side chains as stick models (D419, D427, P429, Y430, S431). On the right panel, the residues from L2 (E280, N281, I282) and L4 (L447, S449, D450), likely to contact L3*, are shown in stick models.

C) The region outlined with a green dashed-line rectangle in panel A (bottom view) is magnified, showing the L1 loops from 3 protomers, colored in yellow, blue, and grey, with the residues likely to form inter-RBD contacts displayed only for L1, for clarity (A262, D263, V264, S265, F266, Y267).

Figure S11: RBD loops are functionally important for viral particles production, cell binding and infectivity

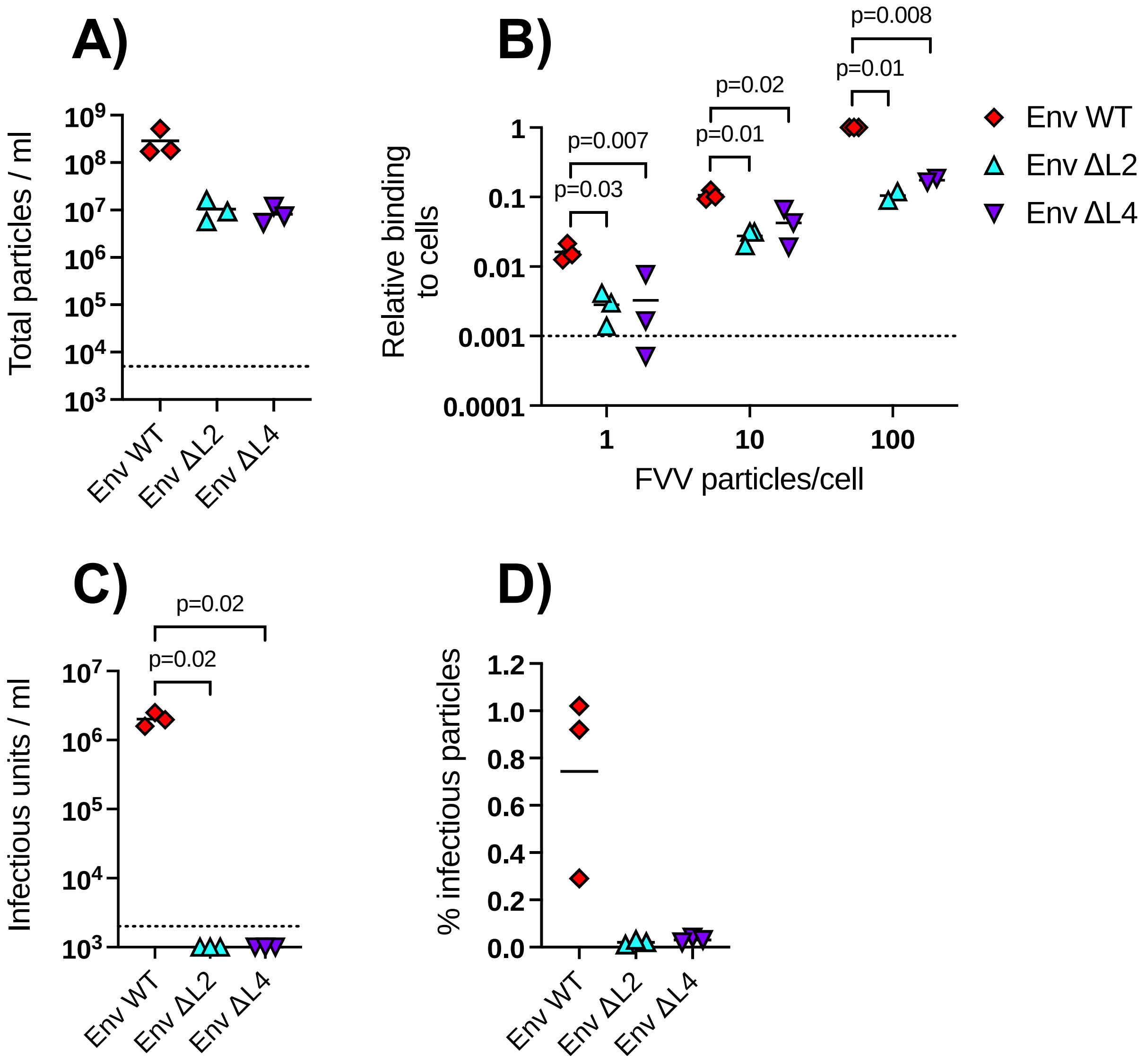

Figure S11: Three batches of FVVs carrying WT or mutated Env were produced. Horizontal lines represent the mean values from tested FVV batches. The dotted lines represent the quantification threshold. Source data are provided as a Source Data file.

A) The concentration of FVV particles was quantified by RT-qPCR amplification. Each batch was titrated two times and the mean titers are presented.

B) FVVs carrying WT and mutated Env were incubated with HT1080 cells on ice for 1 h before washing and quantification of the remaining vector particles by RT-qPCR. The FVV dose was 1, 10 or 100 FVV particles per cell. Values below the threshold were arbitrarily set at 0.0005. The values obtained for the mutant and WT FVVs were compared using the two-way paired t test.

C) FVV infectious titers were quantified on BHK-21 cells. Each batch was titrated two times and the mean titer is plottedblack solid horizontal lines represent the mean values from the three FVV batches. The values obtained for the mutant and WT FVVs were compared using the two-way paired t test.

D) The percentage of infectious FVV particles carrying WT, ΔL2 and ΔL4 Env was calculated as the ratio between the number of infectious particles (panel C) and the amount of vector particles obtained by RT-qPCR (panel A).

Figure S12: Recombinant RBD variants remain monomeric in solution

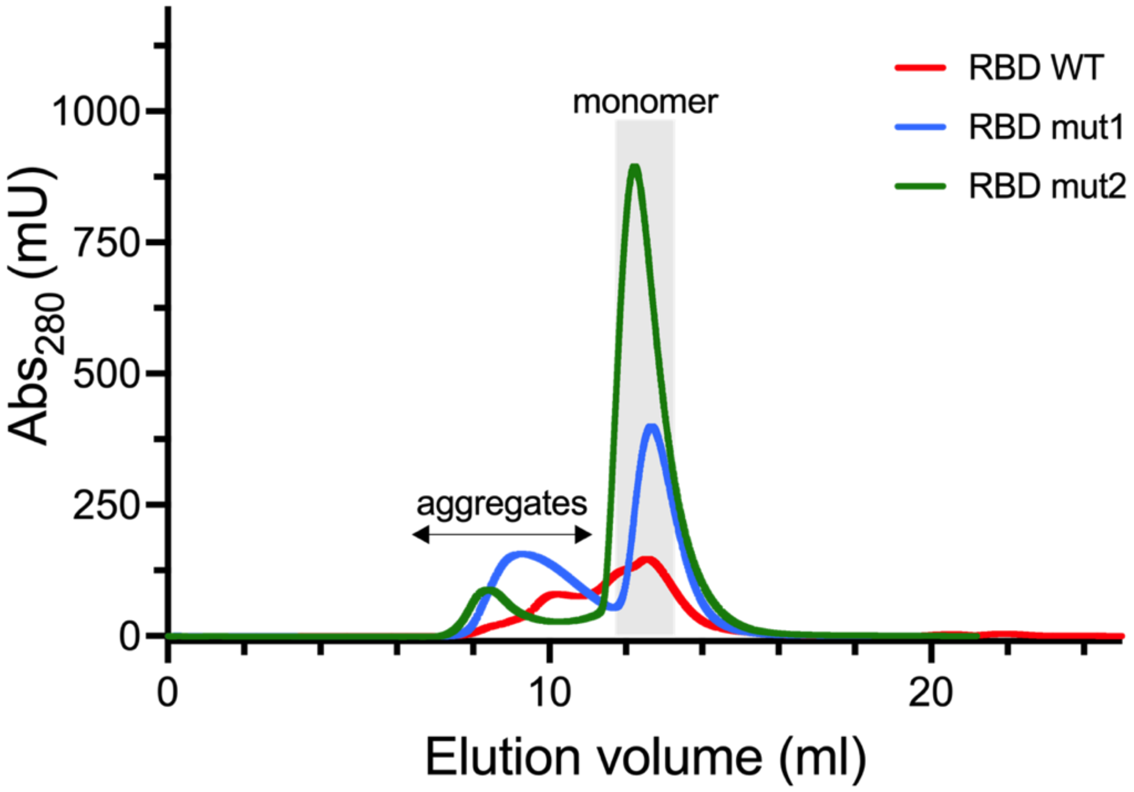

Figure S12 legend: The size exclusion chromatography (Superdex 200) profiles for the GII RBD expressed in mammalian cells are shown for the WT protein (red line) and the variants (blue and green lines).

Figure S13: Flow cytometry gating strategy

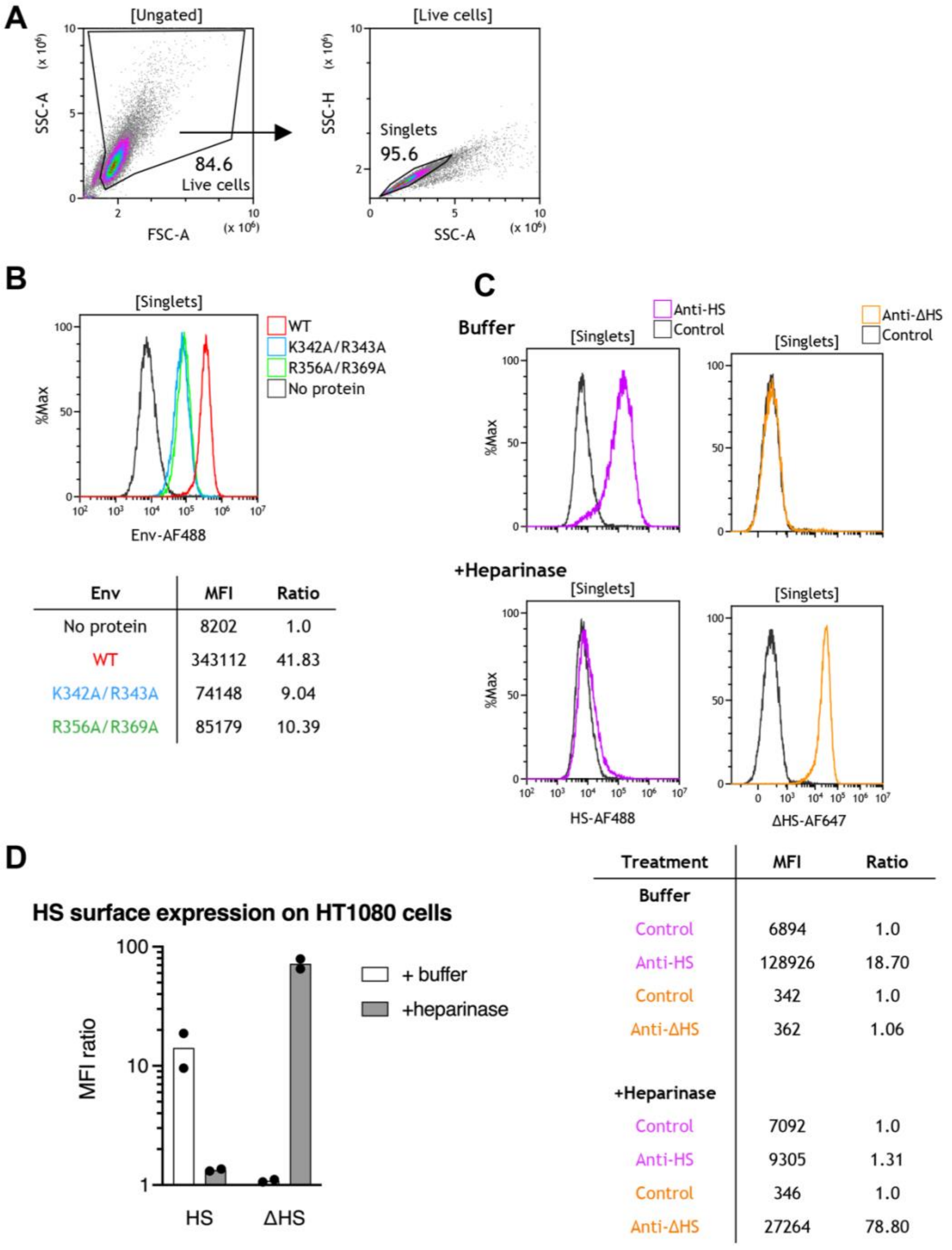

Env	MFI	Ratio
No protein	8202	1.0
WT	343112	41.83
K342A/R343A	74148	9.04
R356A/R369A	85179	10.39

Treatment	MFI	Ratio
**Buffer**		
Control	6894	1.0
Anti-HS	128926	18.70
Control	342	1.0
Anti-ΔHS	362	1.06
**+Heparinase**		
Control	7092	1.0
Anti-HS	9305	1.31
Control	346	1.0
Anti-ΔHS	27264	78.80

Figure S13 legend: Flow cytometry gating strategy for the detection of Env binding and HS expression on SFV-susceptible cell lines. Cells were treated with trypsin-EDTA before labelling with Env proteins or anti-HS antibodies.

A) Representative example of HT1080 single cell selection: live cells were selected by a gate applied on an FSC-A/SSC-A dot-plot and a single cell gate applied on an SSC-A/SSC-H dot-plot.

B) Representative example of Env binding analysis. HT1080 cells were labelled with WT, K342A/R343A (mut1) or R356A/R369A (mut2) ectodomain proteins, StrepMAB-Classic-HRP and anti-HRP-AF488 antibodies. Staining obtained on gated live single cells is presented on the

histogram overlay: MFI is presented on the x-axis and frequency is expressed as the normalized percentage of gated events on the y-axis (%Max). Cells labelled with secondary antibodies only ("control" condition, black curve) were used as a reference. Env-specific staining was quantified by the ratio of MFI from Env treated to untreated cells.

C) Representative example of heparan sulfate staining after treatment with heparinase III. HT1080 cells were treated with heparinase III or buffer and stained with the F58-10E4 antibody specific for heparan sulfate (anti-HS) and the F69-3G10 antibody specific for glycans exposed after heparan sulfate removal (anti-ΔHS). Staining obtained on gated live single cells are presented on the histogram overlay: MFI is presented on the x-axis and frequency is expressed as the normalized percentage of gated events on the y-axis (%Max). Cells labelled with secondary antibodies only ("control" condition, black curve) were used as a reference; HS and ΔHS-specific staining was quantified by the ratio of MFI from labelled to control cells.

D) HT1080 cells were treated with heparinase III or buffer and stained with antibodies specific for HS (HS) or glycans exposed after heparan sulfate removal (ΔHS). Expression levels are calculated as the ratio of MFI from labelled to unlabeled cells (Fig. S13C). Mean values from two independent experiments are shown. Source data are provided as a Source Data file.

Figure S14: Effect of mutations on FVV release and infectious titer

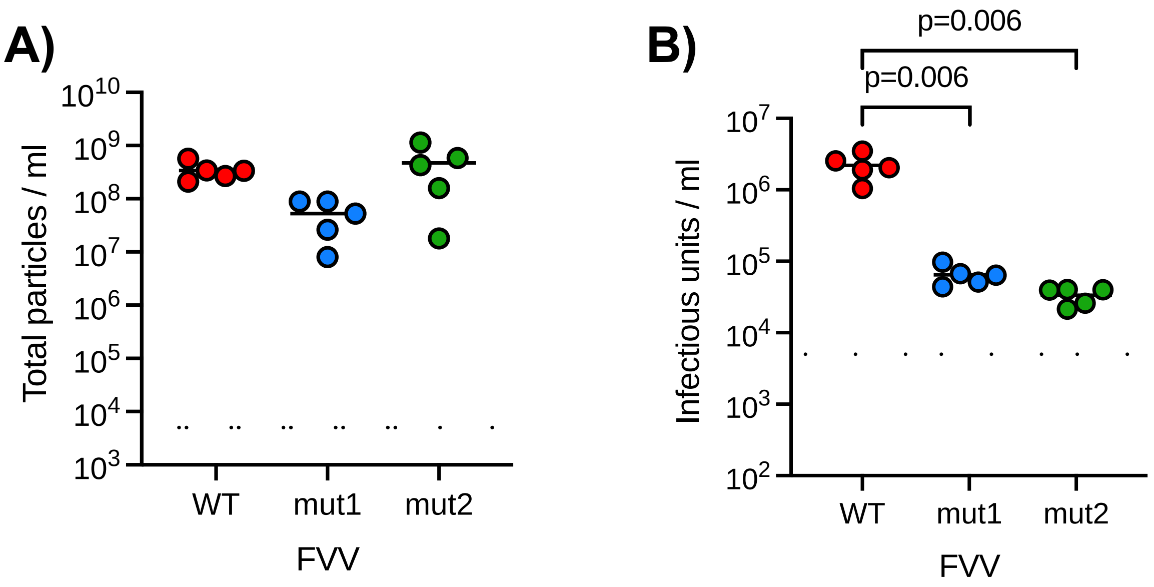

Figure S14 legend: Five batches of FVVs carrying WT (red), mut1 (blue) and mut2 (green) Env were produced, each represented with a single dot.

A) The concentration of the vector particles was quantified by RT-qPCR of β-galactosidase transgene. Each batch was titrated twice, and mean titers are plotted; solid black, horizontal lines represent mean values from the five FVV batches. The dotted black horizontal line represents the quantification threshold. Source data are provided as a Source Data file.

B) FVVs infectious titers were quantified on BHK-21 cells. Each batch was titrated twice, and mean titers are plotted; black solid lines represent mean values from the five FVV batches. The dotted line represents the quantification threshold. The values obtained for the mutant and WT FVVs were compared using the two-way paired t test. Source data are provided as a Source Data file.

Figure S15: Structural basis for RBDjoin region being dispensable for binding to cells

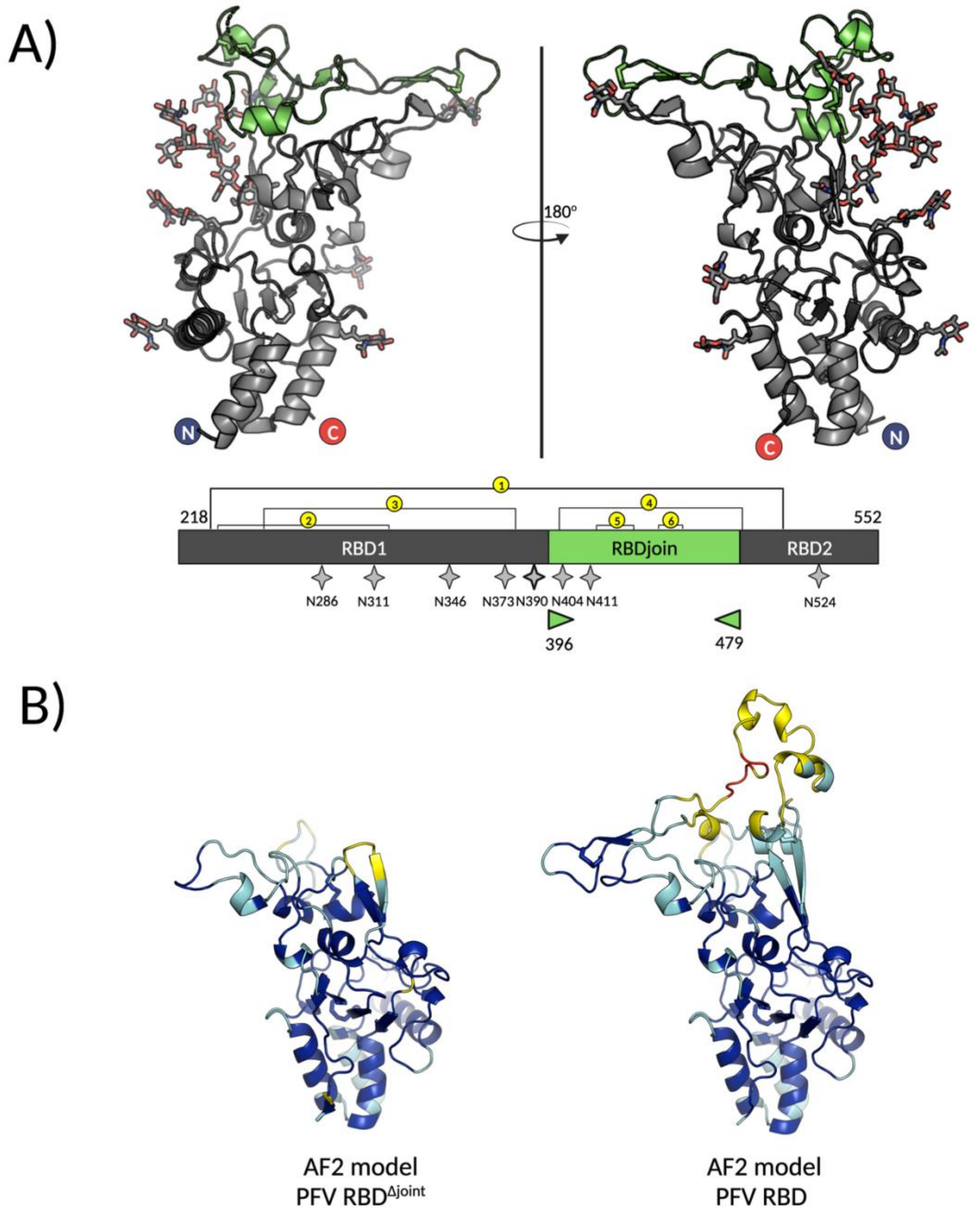

Figure S15: Functional features plotted on the RBD structure.

A) The regions identified in the bipartite PFV RBD as essential [17] (indicated as RBD1 and RBD2 according to the more recent nomenclature [18]) and non-essential (or RBDjoin [18]) for SFV entry are colored in dark grey and green, respectively, and plotted on the X-ray structure of gorilla SFV RBD. The numbering corresponds to the gorilla GII RBD.

B) The AF2 models of the PFV RBD lacking the non-essential RBDjoin region (left panel) and of the whole PFV RBD (right panel) are colored according to the pLDDT values, using the same palette as in Fig. S8.

References

1 Klose, D. P., Wallace, B. A. & Janes, R. W. 2Struc: the secondary structure server. *Bioinformatics* **26**, 2624-2625, doi:10.1093/bioinformatics/btq480 (2010).

2 Ferruz, N., Schmidt, S. & Hocker, B. ProteinTools: a toolkit to analyze protein structures. *Nucleic Acids Res* **49**, W559-W566, doi:10.1093/nar/gkab375 (2021).

3 The PyMOL Molecular Graphics System (DeLano Scientific, San Carlos, CA, USA, 2002).

4 Luftenegger, D., Picard-Maureau, M., Stanke, N., Rethwilm, A. & Lindemann, D. Analysis and function of prototype foamy virus envelope N glycosylation. *J Virol* **79**, 7664-7672, doi:10.1128/JVI.79.12.7664-7672.2005 (2005).

5 Madeira, F. *et al.* Search and sequence analysis tools services from EMBL-EBI in 2022. *Nucleic Acids Res*, doi:10.1093/nar/gkac240 (2022).

6 Robert, X. & Gouet, P. Deciphering key features in protein structures with the new ENDscript server. *Nucleic Acids Res* **42**, W320-324, doi:10.1093/nar/gku316 (2014).

7 Richard, L. *et al.* Cocirculation of two *env* molecular variants, of possible recombinant origin, in gorilla and chimpanzee simian foamy virus strains from Central Africa. *Journal of virology* **89**, 12480-12491, doi:10.1128/jvi.01798-15 (2015).

8 Galvin, T. A., Ahmed, I. A., Shahabuddin, M., Bryan, T. & Khan, A. S. Identification of Recombination in the Envelope Gene of Simian Foamy Virus Serotype 2 Isolated from Macaca cyclopis. *Journal of virology* **87**, 8792-8797, doi:10.1128/JVI.03555-12 (2013).

9 Aiewsakun, P. *et al.* Modular nature of simian foamy virus genomes and their evolutionary history. *Virus Evol* **5**, vez032, doi:doi: 10.1093/ve/vez032 (2019).

10 Krissinel, E. & Henrick, K. Inference of macromolecular assemblies from crystalline state. *J Mol Biol* **372**, 774-797, doi:10.1016/j.jmb.2007.05.022 (2007).

11 Jumper, J. *et al.* Highly accurate protein structure prediction with AlphaFold. *Nature* **596**, 583-589, doi:10.1038/s41586-021-03819-2 (2021).

12 Dong, R., Pan, S., Peng, Z., Zhang, Y. & Yang, J. mTM-align: a server for fast protein structure database search and multiple protein structure alignment. *Nucleic Acids Res* **46**, W380-W386, doi:10.1093/nar/gky430 (2018).

13 Dong, R., Peng, Z., Zhang, Y. & Yang, J. mTM-align: an algorithm for fast and accurate multiple protein structure alignment. *Bioinformatics* **34**, 1719-1725, doi:10.1093/bioinformatics/btx828 (2018).

14 Zhang, Y. & Skolnick, J. Scoring function for automated assessment of protein structure template quality. *Proteins* **57**, 702-710, doi:10.1002/prot.20264 (2004).

15 Effantin, G. *et al.* Cryo-electron microscopy structure of the native prototype foamy virus glycoprotein and virus architecture. *PLoS Pathog* **12**, e1005721, doi:10.1371/journal.ppat.1005721 (2016).

16 Pettersen, E. F. *et al.* UCSF Chimera--a visualization system for exploratory research and analysis. *J Comput Chem* **25**, 1605-1612, doi:10.1002/jcc.20084 (2004).

17 Duda, A., Luftenegger, D., Pietschmann, T. & Lindemann, D. Characterization of the prototype foamy virus envelope glycoprotein receptor-binding domain. *J Virol* **80**, 8158-8167, doi:10.1128/JVI.00460-06 (2006).

18 Lambert, C. *et al.* Potent neutralizing antibodies in humans infected with zoonotic simian foamy viruses target conserved epitopes located in the dimorphic domain of the surface envelope protein. *PLoS Pathog* **14**, e1007293, doi:10.1371/journal.ppat.1007293 (2018).
